# Effectiveness and safety of music therapy for insomnia disorder patients: A protocol for systematic review and meta-analysis

**DOI:** 10.1097/MD.0000000000026399

**Published:** 2021-07-02

**Authors:** Jie Ding, Tianqi Huang, Jinyu Hu, Fuqiang Yuan

**Affiliations:** a College of Music, Jiangxi Science and Technology Normal University; b College of Acupuncture-Moxibustion and Tuina, Jiangxi University of Chinese Medicine; c The Affiliated Hospital of Jiangxi University of Chinese Medicine, Nanchang, China.

**Keywords:** insomnia disorder, music therapy, protocol, systematic review

## Abstract

**Background:**

Music therapy has been widely used clinically to relieve insomnia disorder patients. However, the efficacy of music therapy in the treatment of insomnia disorder patients is uncertain. The purpose of this study is to determine the effectiveness and safety of music therapy in the treatment of insomnia disorder patients.

**Methods:**

Search PubMed, Cochrane Library, Embase, China National Knowledge Infrastructure Database, Wanfang Database, China Science and Technology Journal Database, China Biomedical Literature Database, and search-related randomized controlled trials. Two reviewers will independently select studies, collect data, and evaluate methodological quality through the Cochrane Deviation Risk Tool. Revman V.5.3 will be used for meta-analysis.

**Results:**

This study will evaluate the current status of music therapy treatment for insomnia disorder patients, aiming to illustrate the effectiveness and safety of music therapy treatment.

**Conclusion:**

This study will provide a basis for judging whether music therapy is effective in treating insomnia disorder patients.

**INPLASY registration number:**

INPLASY202150087.

## 1 Introduction

Insomnia is a condition characterized by both nocturnal and diurnal symptoms, one of the most prevalent health concerns in the population and in clinical practice.^[1]^ The main clinical characterized by difficulty falling asleep or difficulty maintaining sleep; frequent awakenings, difficulty returning to sleep after awakenings, or awakening too early with inability to return to sleep; and is accompanied by fatigue, decreased energy, mood disturbances, and reduced cognitive functions, such as impaired attention, concentration, and memory below.^[2–4]^ The prevalence of insomnia symptoms in the general worldwide prevalence ranges from 35% to 50%,^[5]^ and the prevalence of insomnia disorder ranges from 12% to 20%,^[6]^ and according to epidemiological studies show that the prevalence among the adolescent population is increasing. Insomnia with its effects on quality of life, occupational functioning, and physical and psychological health means that the disorder inflicts a heavy burden on individuals and the broader community.^[7]^

The etiology and pathophysiology of insomnia involve genetic, environmental, behavioral, and physiological factors culminating in hyperarousal. Study shows that insomnia is more prevalent in women than in men and is also more commonly diagnosed in people with medical or psychiatric disorders than in the general population.^[7]^ Insomnia treatment includes 2 broad categories, cognitive-behavioral treatment and medication treatment.^[8]^ Treatment for patients with insomnia who do not have a co-occurring illness should begin with patient education regarding sleep hygiene and education to improve sleep.^[9]^ Hypnotic medications are also efficacious, but the long-term side effects of sedative, hypnotic drug use are obvious.^[10]^

Music-supported therapy (MST) is a promising new treatment,^[11]^ and extensive research suggests that music therapy increased the sleep quality of insomnia disorder patients. Music easily elicits movements that stimulate interactions between the perception and action systems, insomnia patients may sensitive to the music, and listening to music has been considered as a therapeutic strategy for insomnia patients’ treatment.

The focus of this study is the efficacy of music therapy for insomnia disorder patients. Therefore, we conducted this study to systematically evaluate the impact of music therapy on insomnia disorder patients. It can provide a basis for the diagnosis and treatment of MST for insomnia disorder.

## 2 Methods

### 2.1 Inclusion criteria for study selection

#### 2.1.1 Types of studies

All randomized controlled trials (RCTs) of music therapy for insomnia disorder patients will be included without language restriction. Non-RCTs, observational studies, cross-over studies, uncontrolled trials, animal trials, and reviews will be excluded.

#### 2.1.2 Types of participants

Inclusion criteria for study populations will be all patients with insomnia disorder. No restrictions will be applied in terms of gender, age, race, condition duration, or intensity.

#### 2.1.3 Types of interventions

##### 2.1.3.1 Experimental interventions

The treatment group will only receive music therapy alone, without any restrictions on music material, type, or treatment process.

##### 2.1.3.2 Control interventions

The control group will receive internationally recognized therapy such as pharmacological therapies. Placebo, no treatment, and sound wave will also be included. Studies that compare the effect of different types of music will be excluded.

#### 2.1.4 Types of outcome measures

##### 2.1.4.1 Primary outcomes

Sleep quality will be accepted as the primary outcomes, measured by the Pittsburgh Sleep Quality Index; can also be evaluated using the Athens Insomnia Scale.

##### 2.1.4.2 Additional outcomes

The sleep onset latency (in minutes) and sleep efficiency (%) and safety assessment will be considered a secondary outcome.

### 2.2 Search methods for the identification of studies

#### 2.2.1 Electronics searches

The following electronic databases will be searched: PubMed, Embase, the Cochrane Library, the China National Knowledge Infrastructure, Chinese Science and Technology Periodical Database, Wanfang Database, and Chinese Biomedical Literature Database. We will search the databases from the beginning to January, 2021. Search terms consist of disease (disorders of initiating and maintaining sleep, early awakening, primary insomnia, sleep initiation dysfunction, and chronic insomnia); intervention (music therapy, acoustic stimulation, and music-supported therapy); and research types (randomized controlled trial, controlled clinical trial, and random trials). The PubMed search strategy is shown in Table 1.

**Table 1:** Search strategy used in PubMed database.

Number	Search items
#1	Randomized controlled trial [pt]
#2	Controlled clinical trial [pt]
#3	Randomized [tiab]
#4	Clinical trials as topic [mesh: noexp]
#5	Randomly [tiab]
#6	Trial [ti]
#7	OR/ #1–#7
#8	Animals [mh] NOT humans [mh]
#9	#7 NOT #8
#10	Disorders of Initiating and Maintaining Sleep [Mesh]
#11	Early Awakening [All Fields)
#12	Nonorganic Insomnia [All Fields)
#13	Primary Insomnia [All Fields)
#14	Transient Insomnia [All Fields)
#15	Rebound Insomnia[All Fields)
#16	Secondary Insomnia [All Fields)
#17	Sleep Initiation Dysfunction [All Fields)
#18	Sleeplessness[All Fields)
#19	Insomnia Disorder[All Fields)
#20	Chronic Insomnia[All Fields)
#21	Psychophysiological Insomnia[All Fields)
#22	OR#10-#21
#23	Music Therapy [Mesh]
#24	Acoustic Stimulation [All Fields)
#25	Music-supported therapy [All Fields)
#26	OR/ #23–#25
#27	#9 AND #22 AND #26

#### 2.2.2 Search for other resources

We will also retrieve the relevant conference papers, and search for new trials related to music therapy treatment on mood in post-stroke rehabilitation patients on the WHO International Clinical Trials Registration Platform and the Clinical Trials.gov.

### 2.3 Data collection and analysis

#### 2.3.1 Selection of studies

**Figure 1:**
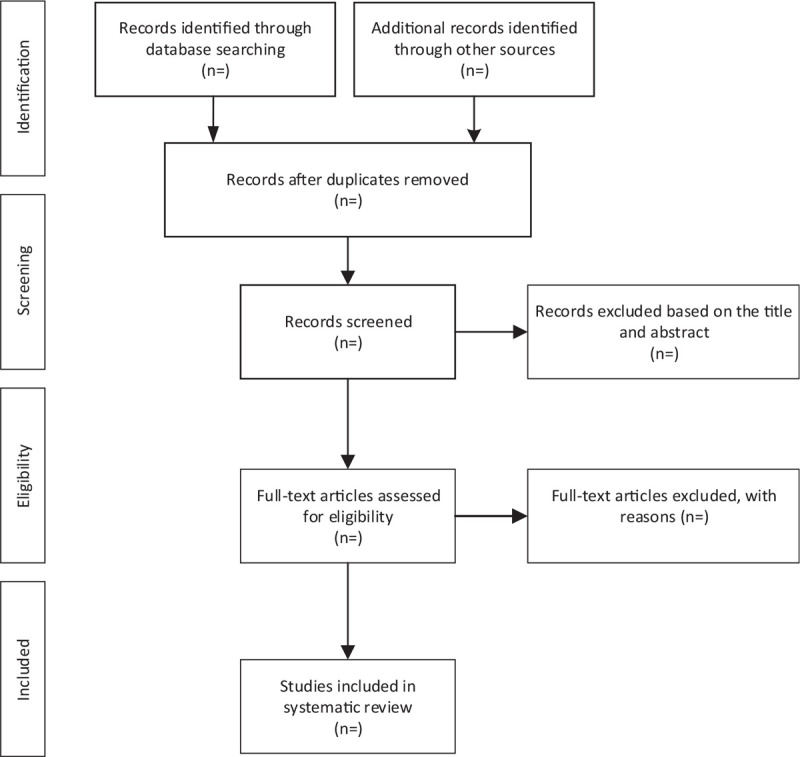
Flow diagram of the study selection process.

We will import the retrieved literature into EndNote X7 software and delete the duplicate data. After that, 2 reviewers will independently scan the titles and abstracts. Unrelated literature will be deleted. If they cannot determine whether to include the study, they will obtain the full text of the article for judgment. Two reviewers will independently evaluate the eligibility of these articles based on inclusion and exclusion criteria. Any disagreements will be resolved through group discussions. The study selection procedure is shown in Figure 1.

#### 2.3.2 Data extraction and management

The data extraction for eligible studies will be completed independently by 2 authors, and any disagreement will be resolved through discussion with the third author. The extracted data will mainly include the first author, time of publication, patient characteristics, sample size, interventions, follow-up period, outcome measures, and adverse events. If necessary, we will try to contact the author for the details by email.

### 2.4 Risk of bias assessment

Two independent authors will evaluate the risk of bias among the final included studies using the risk of bias assessment tool by the Cochrane collaboration.^[12]^ The contents will include (1) random sequence generation; (2) allocation concealment; (3) blinding of participants and personnel; (4) blinding of outcome assessment; (5) incomplete outcome data; (6) selective reporting; and (7) other sources of bias. Each study will be evaluated as high, low, or unclear risk of bias for each item. Discrepancies will be resolved through further discussion with the third author.

### 2.5 Quantitative data synthesis and statistical methods

#### 2.5.1 Quantitative data synthesis

We will conduct statistical analysis through RevMan 5.3 software. For categorical data, we will calculate with the risk ratio and 95% confidence intervals (CIs). For continuous variables, mean difference (MD) will be included in the meta-analysis. If outcome variables are measured on different scales, results will be reported as standardized MDs with 95%CI.

#### 2.5.2 Assessment of heterogeneity

We will use chi-square test and *I*^2^ test to evaluate the statistical heterogeneity. When *P* > .10 and *I*^2^ ≤ 50%, the research results will not be considered heterogeneous; otherwise, it will be considered heterogeneous.

#### 2.5.3 Assessment of reporting biases

When more than 10 studies are included, a funnel plot will be generated to detect the reporting bias. In addition, we will use the Egger test to check the asymmetry of the funnel plot.

#### 2.5.4 Subgroup analysis

If the included studies have significant heterogeneity, we will perform subgroup analysis based on different control groups.

#### 2.5.5 Sensitivity analysis

When sufficient studies are available, sensitivity analysis will be used to assess the robustness of the meta-analysis based on methodological quality, sample size, and missing data.

#### 2.5.6 Grading the quality of evidence

We will assess the quality of evidence by the Grading of Recommendations Assessment, Development, and Evaluation and rate it into high, moderate, low, or very low 4 levels.^[13–14]^

## 3 Discussion

Music therapy is a non-pharmacological, non-invasive, inexpensive intervention with non-adverse reactions, which can be executed easily and successfully.^[15]^ MST has been widely used in the treatment of insomnia disorder patients, the mechanisms underlying the relationship between music and sleep could be a physiological relaxation response to the music, promoting a reduction of the arousal level, thereby reducing sleeping problem symptoms, it may increase peripheral levels of β-endorphins, promote changes in serotonin levels, and decrease sympathetic activity.^[16]^ but the clinical efficacy of MST has not been scientifically and systematically evaluated. This study aims to evaluate the clinical efficacy and safety of MST in the treatment of insomnia disorder patients. The conclusions of this study can provide evidence-based medicine recommendations for MST treatment of insomnia disorder patients.

Research limitations: first of all, in the process of MST treatment, the choice of treatment, the choice of the treatment site, time, and frequency may be heterogeneous. Second, this study has set strict inclusion criteria, and the inclusion of high-quality literature may have less impact. The reliability of systematic review depends to a large extent on comprehensiveness and methodological quality. Third, the included studies do not limit language types, and there are certain language biases.

## Author contributions

**Conceptualization:** Jinyu Hu, Fuqiang Yuan.

**Data curation:** Jie Ding, Tianqi Huang, Jinyu Hu.

**Formal analysis:** Jie Ding, Jinyu Hu.

**Funding acquisition:** Jie Ding.

**Investigation:** Tianqi Huang, Jinyu Hu, Jie Ding.

**Methodology:** Tianqi Huang, Jinyu Hu.

**Project administration:** Jie Ding, Jinyu Hu.

**Software:** Tianqi Huang, Jinyu Hu.

**Supervision:** Jie Ding, Jinyu Hu.

**Validation:** Jie Ding.

**Visualization:** Jinyu Hu.

**Writing – original draft:** Jie Ding, Jinyu Hu.

**Writing – review & editing:** Fuqiang Yuan, Jinyu Hu.
